# Modeling horizontal gene transfer (HGT) in the gut of the Chagas disease vector *Rhodnius prolixus*

**DOI:** 10.1186/1756-3305-4-77

**Published:** 2011-05-14

**Authors:** Scott Matthews, Vadrevu SreeHari Rao, Ravi V Durvasula

**Affiliations:** 1 Department of Internal Medicine, University of New Mexico and Raymond G. Murphy VA Medical Center, 1501 San Pedro Dr SE. Albuquerque, NM, 87108, USA; 2 Jawaharlal Nehru Technical University, Hyderabad, India

## Abstract

**Background:**

Paratransgenesis is an approach to reducing arthropod vector competence using genetically modified symbionts. When applied to control of Chagas disease, the symbiont bacterium *Rhodococcus rhodnii*, resident in the gut lumen of the triatomine vector *Rhodnius prolixus *(Hemiptera: Reduviidae), is transformed to export cecropin A, an insect immune peptide. Cecropin A is active against *Trypanosoma cruzi*, the causative agent of Chagas disease. While proof of concept has been achieved in laboratory studies, a rigorous and comprehensive risk assessment is required prior to consideration of field release. An important part of this assessment involves estimating probability of transgene horizontal transfer to environmental organisms (HGT). This article presents a two-part risk assessment methodology: a theoretical model predicting HGT in the gut of *R. prolixus *from the genetically transformed symbiont *R. rhodnii *to a closely related non-target bacterium, *Gordona rubropertinctus*, in the absence of selection pressure, and a series of laboratory trials designed to test the model.

**Results:**

The model predicted an HGT frequency of less than 1.14 × 10^-16 ^per 100,000 generations at the 99% certainty level. The model was iterated twenty times, with the mean of the ten highest outputs evaluated at the 99% certainty level. Laboratory trials indicated no horizontal gene transfer, supporting the conclusions of the model.

**Conclusions:**

The model treats HGT as a composite event, the probability of which is determined by the joint probability of three independent events: gene transfer through the modalities of transformation, transduction, and conjugation. Genes are represented in matrices and Monte Carlo method and Markov chain analysis are used to simulate and evaluate environmental conditions. The model is intended as a risk assessment instrument and predicts HGT frequency of less than 1.14 × 10^-16 ^per 100,000 generations. With laboratory studies that support the predictions of this model, it may be possible to argue that HGT is a negligible consideration in risk assessment of genetically modified *R. rhodnii *released for control of Chagas disease.

## Background

The challenge of controlling vector borne diseases has fueled development of novel strategies that involve genetic manipulation of arthropod vectors. The goal of these strategies is reduction of vector competence, the ability of an arthropod vector to transmit an infectious pathogen. We have described a method, termed paratransgenesis, that involves modification of symbiotic bacteria of an arthropod [1-3]. In this approach a symbiotic bacterium is transformed with foreign genes expressing anti-pathogen agents. We have validated this approach in a control system for Chagas disease involving the triatomine vector *Rhodnius prolixus *(order *Hemiptera*, family *Reduviidae*) and its obligate symbiont, *Rhodococcus rhodnii *[4]. *R. rhodnii *resides in the vector gut lumen, in close proximity to *Trypanosoma cruzi*, the causative agent of Chagas disease. In our system *R. rhodnii *is genetically modified to export an insect immune peptide, cecropin A, at concentrations sufficient to kill *T. cruzi*.

Implementation of this approach to Chagas disease control would involve release of genetically modified bacteria. To target field population of triatomines we have developed a synthetic fecal paste, CRUZIGARD, simulating natural coprophagic modes of symbiont dispersal. We have demonstrated efficacy of this approach in close-caged trials [5] and are currently testing it in a simulated greenhouse environment.

Environmental release of transgenic bacteria merits stringent risk assessment to address significant concerns raised by release of genetically modified bacteria. An important part of this framework involves evaluating the probability of foreign genetic material migrating to environmental organisms.

Horizontal gene transfer (HGT) is the most likely migration route for foreign genetic material, e.g. the cecropin A transgene. HGT has played a prominent role in bacterial evolution and is implicated in phenomena impacting human health, including antimicrobial resistance and virulence regulation. Several studies have established the influence of horizontal gene transfer on the composition of prokaryotic genomes [6,7]. Lawrence and Ochman used variations in codon bias to estimate that approximately 18% of the *Escherichia coli *K-12 genome had been acquired through horizontal gene transfer [8]. The constancy in the size of the *E. coli *genome suggests gene acquisition balanced by gene loss.

Genes acquired through horizontal transfer are thought to confer a selective advantage. Studies of gene transfer in the environment and under simulated environmental conditions have examined HGT under selection pressure [9,10]. In the first part of our risk assessment, we present a theoretical model predicting HGT in the gut of the vector *R. prolixus *from the genetically transformed symbiont *R. rhodnii *to a closely related non-target bacterium in the absence of selection pressure. The non-target bacterium in this model, *Gordona rubropertinctus*, has been recovered from the gut of *R. prolixus *and another triatomine Chagas disease vector, *Triatoma dimidiata *[11].

The non-target bacterium and the model locus, the gut of the arthropod vector, were selected because they compose a system with parameters regarded as favorable to HGT: a high degree of relatedness between the putative donor (*R. rhodnii*) and recipient (*G. rubropertinctus) *bacteria, temperatures between 26-30°C, nutrient enrichment, and close and constant proximity of donor and recipient organisms [12-16]. The arthropod gut has received attention as a "hot spot" for horizontal gene transfer and is likely the environmental locus most favorable to HGT [9]. The second part of our assessment involves laboratory experiments under conditions exaggerated to favor HGT. The objective of these experiments is to establish limits of probability for transgene horizontal gene transfer (tHGT) following release of genetically transformed *R. rhodnii*. Results of these experiments have the potential to validate the model and extend its conclusions.

### Theoretical Model

In this model both donor and recipient organisms are gram-positive actinomycete bacteria. We have drawn upon studies of horizontal gene transfer in gram-positive bacteria, principally *B. subtilis *and *S. pneumoniae*, to construct schematics for HGT between *R. rhodnii *and *G. rubropertinctus *[17,18]. We have defined the outcome of interest for the model and the adverse event for risk assessment as integration of cecropin A transgene into recipient bacteria. The model addresses HGT as a composite event whose probability is determined by the joint probability of independent events. Here, independent events are the gene transfer modalities of transformation, transduction, and conjugation. In turn, each of these processes may be viewed as composite events the probability of which is determined by the probability of joint independent serial events.

Assumptions incorporated in the model reflect parameters identified in previous studies as most favorable to HGT. Where possible, these parameters have been used in governing equations for the model. The following are the underlying assumptions and a list of values for various parameters in the model equations:

• The genome of *R. rhodnii *has a high degree of similarity to that of *Mycobacterium tuberculosis*, sufficient to enable heterologous transformation

• *R. rhodnii *reaches a population maximum of 10^8 ^colony forming units (CFU) 5-7 days after an *R. prolixus *bloodmeal, declining to 10^5 ^CFU during starvation

• The *R. prolixus *gut is a sphere with axial symmetry and volume of 0.2 - 0.3 mL

• *G. rubropertinctus *population during competence is 10^8 ^CFU/mL

• *G. rubropertinctus *is continuously competent

• *G. rubropertinctus *is a sphere with radius 1 × 10^-4 ^cm

• *R. rhodnii *chromosomal fragment (cell-free DNA) is viable for transformation for a period of twenty minutes

• *R. prolixus *gut is isothermic at 27°C

• The mean viscosity of bloodmeal in the *R. prolixus *gut is 0.48 g/cc

• *R. prolixus *feeds to repletion once per 36 days, without interruption

• *R. rhodnii *DNA fragment behaves as an ellipsoid in fluid

• Cell-free *R. rhodnii *DNA is viable for a period of twenty minutes following cell lysis

• *G. rubropertinctus *transformasome binding of *R. rhodnii *cell-free DNA is non-competitive

• Rate of gene transfer for the recipient bacteria is estimated from the rate assigned to *Escherichia coli*. *E. coli *is considered to have the highest rate of horizontal gene acquisition of any sequenced terrestrial bacterium [19]. This rate is estimated at 16 kb/million years, suggesting one-fourth of the organism's genome has been acquired through horizontal transfer. Using codon usage bias patterns identified by Muto and Osawa [20] to set an upper limit for horizontal transfer, our model assumes that during this million-year period 1,160 kb - one fourth of the genome of the organism - were transiently acquired through horizontal transfer [8,21,22]. This is equivalent to saying 72 kb were transferred for every 1 kb persisting in the organism.

• Between fifteen and twenty percent of genes in the donor bacterium may be classified as genes conferring selective advantage to the recipient bacterium. This figure is derived from the rate of gene transfer calculated above for *E. coli*.

### Governing equations

Mean path length, light particle among heavy particles:

Collision frequency, light particle among heavy particles:

Probability of composite event A denoted by P(A), where A is decomposable into independent events B, C, and D:

Integration of a vector gradient:

where R is the region of integration in the xy rectangle.

Lotka-Volterra equations:

where D is the donor bacterium, R is the recipient bacterium, P is the phage, r is the growth rate of the donor bacterium, and a is the growth rate of the recipient bacterium.

### Modeling Transformation

This study uses the following seven-step schematic for HGT through transformation:

1. Lysis of transformed *R. rhodnii *cells in the *R. prolixus *gut

2. Diffusion to binding sites on external membrane of *G. rubropertinctus*; model uses Monte Carlo method for selection per iteration of 10 - 40 binding sites

3. Binding of DNA fragments ranging from 18 to 40 kb, with 18 kb and 32 kb being the modes in a bimodal distribution

4. During *G. rubropertinctus *cytoplasm intake, the bound fragment is "nicked" at various points along its length. The modal nicks occur at 6 kb and 8.5 kb intervals and fragment size ranges from 5-10 kb

5. Fragments integrate linearly into recipient DNA as a single strand. Integration involves a replacement reaction, wherein a recipient strand leaves and a donor strand takes its place. This replacement reaction is driven by homology of the single strand donor DNA to recipient chromosome and facilitated by *RecA *equivalents in recipient cells

6. The *R. rhodnii *single strand invades the recipient DNA duplex and, through *RecA *mediated recognition, finds homology and is integrated, with corresponding removal of relevant recipient DNA strand

**7**. The integrated donor strand creates a mismatched heteroduplex, resolved by DNA replication or repair

The sixth step circumscribes our outcome of interest, integration of an *R. rhodnii *fragment containing the cecropin A transgene into *G. rubropertinctus*. Model operations described below are keyed to steps 1-6 of this schematic:

1,2. In modeling binding of cell-free *R. rhodnii *DNA, we assume *R. rhodnii *mortality follows stationary phase kinetics during the competence interval of *G. rubropertinctus *and calculate probability that cell-free *R. rhodnii *DNA makes contact with a competent *G. rubropertinctus *cell using the equation

where z is the frequency of collision between a light particle (cell-free *R. rhodnii *DNA) and a heavy particle (competent *G. rubropertinctus *cell), v is the velocity of the *R. rhodnii *cell free DNA in the gut lumen, t is the time interval during which the cell-free DNA is viable, and λ is the mean free path of the *R. rhodnii*. Using v = 9.05 × 10-10 cm, t = 1200 seconds (20 minutes), λ = 7.0 × 10^-16^, and dividing the number of putatively competent *G. rubropertinctus *cells in the gut lumen (10^8^) by the collision frequency value for the total number of cells with adsorbent capacity in the gut lumen adjusted per area, 10^12^, produces an estimate of the probability of contact of *G. rubropertinctus *cells with *R. rhodnii *cell-free DNA.

3. The model calculates the probability any gene would, by random assortment, be included in the 18 - 40 kb fragments bound to the membrane. Since *M. tuberculosis *is thought to be similar in size and composition to *R. rhodnii*, the sequenced *M. tuberculosis *genome (4,411,532 bp) is used to approximate the size of the *R. rhodnii *genome. This similarity has proved sufficient to permit development of heterologous transformation elements [5,23]. Using Monte Carlo method with modes in a Gaussian distribution set at 18 and 36 kb, we compute as n!(4,411,532)/18 - n!(4,411,532)/36) the probability that any single gene equivalent in length to the 4200 bp of cecropin A is included in one of these bound fragments

4. Size of donor fragments is represented by a symmetrical matrix, with each base pair assigned a horizontal and a vertical element in the matrix. Size of *R. rhodnii *cell-free DNA fragments after intake into the cytoplasm will vary from 5-10 kb. The model determines size of these fragments using Monte Carlo method with a range of 5-10 kb and modes set at 6 and 8.5 kb. If, for example, the Monte Carlo evaluation determined a fragment size of 9 kb, the model would generate a 9,000 by 9,000 matrix

5, 6. Matrix composition and matrix operations determine which fragments - and hence which genes - are integrated into the recipient organism. Gene candidates for inclusion are evaluated as vectors. Each gene has co-terminal and coincident column and horizontal vectors. Termination sequences or "cut points" are inserted at random points in the row and column vectors. These provide "stop" signals for evaluation of the vector, and represent length, in base pairs, of the gene. The length of each gene varies from 560 bp to 6200 bp, per the *M. tuberculosis *genome, and is assigned per iteration using Monte Carlo method. Each base pair is randomly assigned a value between -1 and 1. The value of the scalar for the vectors is never more than 1 or less than -1

The column vector assigned each gene in the matrix represents fitness contribution of that gene. Positive fitness contribution genes have vector sums equal to or approaching 1. Fifteen to twenty percent of donor bacterium genes are designated "positive fitness contribution genes", indicating they would confer increased fitness upon the recipient bacterium. The remaining seventy-five to eighty percent of donor bacterium genes are designated fitness-neutral genes or non-coding DNA segments. Fitness-neutral genes have column vector sums with asymptotic minima at 0 and asymptotic maxima at 1. Non-coding DNA segments are represented by column vector sums between 0 and -1. Positive fitness genes may be followed immediately in the matrix by column vectors whose sum is between 0 and -1 or by another positive fitness contribution gene but not by a fitness-neutral gene.

Total value for the vectors is keyed to a rate of 1,160 kb per million years, with variation per iteration determined by Monte Carlo method. Using the Taylor series we scale this rate so the vectors representing each gene have an equivalent chance of being assigned a value of one, regardless of size (number of base pairs).

Using random assortment mediated by Monte Carlo method, positive fitness contribution genes are assigned to sections of the donor chromosome, and hence, to the bound and ingested fragments. Thus, each donor fragment represented by the matrix may contain between zero and four positive fitness contribution genes.

The row vector in the matrix represents homology to recipient DNA required for integration. Here, borrowing from the applications of Markov chains with absorbing states developed by Karlin et al. [24], we designate the homologous segment of the *G. rubropertinctus *recipient chromosome a contiguous three-codon segment [24,25]. This three-codon segment is composed of nine nucleotides nondegenerative in function: should the identity of one of the nucleotides change, homology no longer obtains.

One and only one arrangement of these nine nucleotides is designated the absorbing state. This state indicates homology and triggers the transition, integration into the recipient bacterium. We use Monte Carlo method to develop a Gaussian distribution matching the 15-20% positive fitness contribution genes in the donor organism, i.e., we assume distribution of homologies between donor and recipient organism and numerical incidence of positive fitness contribution genes are complementary.

Constructing a Markov Chain transition matrix using ni1, ni2,.. nii1, as frequencies for each nucleotide, and calculating a mean distance between successive occurrences as 1/ni1 nii1 niii1 allows us to calculate the probability of each state (alignment of nine nucleotides) as well as the absorbing state occurring in a donor strand of DNA. Dimensions of the matrix here are determined by length of the donor DNA strand, as described earlier.

So, scalar value of this second (horizontal) vector may be computed as the frequency or probability of the nine-nucleotide segment occurring in the matrix and represented as the matrix score for this outcome.

For each gene, the vertical and horizontal vectors are added. This results in a vector with x and y components, which themselves compose the vector gradient (y/x). When the integral of the vector gradient after addition of the joint probabilities determined in steps 1 and 2 is equal to or greater than the integral of the inverse of the rate of gene transfer, then the transition is realized and we consider the gene integrated into the recipient chromosome. If the integral of the vector gradient is less than the integral of the rate of gene transfer, then the transition is not realized. In practice, the values of x and y coordinates in the vector gradient must be equal to or very close to 1 for the transition to occur.

When requirements are met for transition of a positive fitness contribution gene, then that gene and the two following genes or non-coding DNA segments also make the transition.

Transition for transgenes is represented as occurring when (1) a fitness neutral gene meets transition requirements between two fitness positive genes making transition independently or (2) a fitness neutral gene has row vectors at the beginning and end of the column vector attaining the absorbing states indicating homology, so that each of these can be integrated with the column vector scalar to yield a value equal to or greater than the integral of the inverse rate of gene transfer.

### Modeling transfer by transduction

The cecropin A transgene is not associated with a mobile genetic element and requires an integrase for transformation in the laboratory. Given that the probability is very low that any naturally occurring suitable integrase would be found in the gut of *R. prolixus*, we have modeled probability of transgene transfer through transduction as the probability of serial error propagations within a Poisson distribution. The model addresses inclusion of transgene sections as errors and evaluates the probability of errors propagated and continued serially using a matrix. Monte Carlo method is used to generate phage population numbers as multiples of the donor/recipient CFU ratios according to the Lotka-Volterra equations adapted by Lenski and Levin [26]. These population sizes determine the size of the matrix, with the frequency of gene integration keyed to the 1, 160 kb/myr rate used in modeling HGT by transformation. Evaluation of all phages in a population constitutes iteration and is regarded as representing a single recipient cell generation. The matrix evaluates probability of these errors as occurring as a column vector and probability of their serial occurrence constituting the transgene in the recipient organism as a row vector. When the matrix has constituted the transgene, integration takes place.

### Modeling transfer by conjugation

Transfer of the transgene through conjugation is also constrained by absence of association with a mobile element. We have modeled transfer by conjugation as the probability of serial error propagations within a Poisson distribution evaluated by a Markov Chain matrix. In these operations the size of the plasmid is not an included parameter; it is simply assumed that, as a result of the error in a Poisson distribution, fragments of transformed *R. rhodnii *will vary in size as per the transformation model and the hypothetical plasmid will be large enough to accommodate these fragments. Similar to modeling of transfer by transduction, we use Monte Carlo method to generate plasmid population sizes as multiples of the donor cell population. The lower limit of the Gaussian distribution used here represents a plasmid copy number of six, with the upper limit varying as multiples of six. Evaluation of all plasmids in a population constitutes iteration and is regarded as representing a single recipient cell generation. As in the operations for modeling transfer by transduction, the matrix evaluates probability of these errors as occurring as a column vector and probability of their serial occurrence constituting the transgene in the recipient organism as a row vector. When the matrix has constituted the transgene, transition takes place and the transgene is regarded as integrated.

### Model output

The model treats HGT as a composite event whose probability is determined by the joint probability of independent events. Here, the independent events are the gene transfer modalities transformation, transduction, and conjugation. In turn, each of these processes may be viewed as composite events whose probability is determined by the probability of joint serial events.

The output of the model may be summarized as the additive probabilities of HGT by transformation, conjugation, and transduction. It is read as a frequency, incidence of HGT per 100,000 generations, and is assigned a certainty value, here 99%. Results reported are the mean of the highest output from any ten iterations. Mathematica 4.2 (Wolfram Research, Inc., Chicago, IL) and Crystal Ball 2000.2, Professional edition (Decisioneering Incorporated, Grand Junction, CO) were used to develop and execute the model.

## Methods

Studies 1 and 2 were conducted under conditions regarded as favoring HGT. These conditions include temperatures between 26-30°C, nutrient enrichment, high levels of relatedness between prospective donor and recipient, and spatial proximity [27,28].

### Study I: *In vitro *incubation

**Figure 1:**
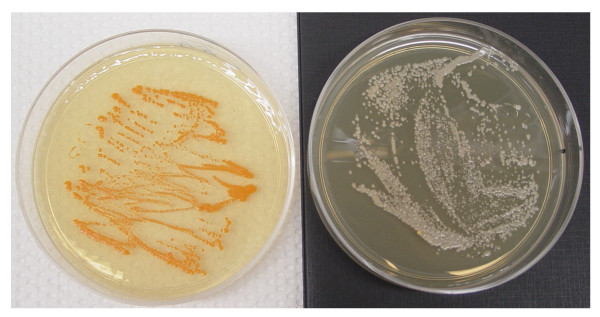
**Contrast in pigmentation between *G. rubropertinctus *and *R. rhodnii *makes the bacteria readily distinguishable when cultured**.

The putative donor bacterium *R. rhodnii *was transformed with the plasmid pRrMDWK6 as per Durvasula et al [1] to express a marker gene conferring resistance to the antibiotic kanamycin and co-incubated in test tubes with *G. rubropertinctus *at exaggerated concentrations. *G. rubropertinctus *is a closely related actinomycete bacterium which, like *R. rhodnii*, has a soil reservoir in the endemic region. There is evidence to suggest G. *rubropertinctus *may have a symbiotic function in the triatomine Chagas vector *T. dimidiata *(Durvasula, R. and Pennington, P., unpublished data). *G. rubropertinctus *also manifests reddish-orange colonies when cultured, making it readily distinguishable from *R. rhodnii*, (Figure 1) and the two bacteria have comparable generation times. Finally, *G. rubropertinctus *is exquisitely resistant to kanamycin, having an MBC of 25 μg/mL.

Clonal populations of *G. rubropertinctus *collected from field sites in Guatemala and *R. rhodnii *(ATCC 35071) transformed with pRrMDWK6 were maintained in BHI medium. Bacterial concentrations were determined using serial dilutions and spectrophotometirc assays [1]. Aliquots of the two bacteria were removed and diluted to a final volume of 2 mL for two incubation sets. The first set simulated bacterial populations in the vector gut lumen. In CFU/mL, donor: recipient (*R. rhodnii *: *G. rubropertinctus*) ratios for these incubations were 10^6^:10^6^, 10^8^:10^6^, and 10^6^:10^8^. The second set exaggerated concentration differentials, simulating conditions represented in the literature as favorable to HGT [27,28]. Donor: recipient (*R. rhodnii *: *G. rubropertinctus) *ratios for these incubations were 10^3^:1, 10^4^:1, 10^6^:1, 10^9^:1, 10^12^:1, and 1:10^6^. Incubations were prepared in duplicate and incubated at 28°C with continuous shaking at 220 rpm.

At intervals of at 24, 48, 96, and 240 hours, 10 μL aliquots were removed from the test tube mixture and plated in duplicate on BHI agar and BHI agar with kanamycin at 50 μg/mL. Dishes were sealed and placed in 28°C incubator for a minimum of 21 days. Following this incubation period, samples were visually inspected for evidence of bacterial growth.

### Study 2: *In vivo *incubation

Study 2 evaluated transfer in the arthropod gut. This study was conducted under sterile conditions. Eggs were removed from laboratory *R. prolixus *colonies and surface sterilized according to methods described in Durvasula and Taneja [29]. Sterilized eggs were transferred to a cage in a sterile incubator set at 27°C. Following eclosure, 10% of the nymphs in each colony were sacrificed and plated on BHI agar to confirm the aposymbiotic state. At intervals of 7 days after eclosure, nymphs were fed to repletion with a blood meal via membrane, containing 10^8 ^CFU/mL *R. rhodnii *and approximately 10 μg of pRrMDWK6 plasmid per mL of solution. These concentrations simulate those of the laboratory electroporation procedures used to transform *R. rhodnii *with the pRrMDWK6 plasmid [30].

Nymphs were returned to the incubator and sacrificed 168 hours after the second feeding. This involved washing the exterior of the bug in phosphate buffered saline (PBS), placing the bug in a microcentrifuge tube with attached top, grinding the bug with plastic pestle to liberate gut contents, and diluting to a final volume of 80 μL with PBS.

Two 30 μL aliquots from each tube were plated on BHI agar and BHI with kanamycin 50 μg/mL. Plates were labeled, sealed, and stored in an incubator at 28°C for 3 weeks. Throughout this period plates were evaluated for growth using visual inspection.

Colonies of *R. rhodnii *were assayed via PCR for the MKα primer, using MKαF5 primers 5'-CTCTCAGAGTTAACTATTCTTTGTACGCC-3' and 5'-GCGAACGCTCCCGCG GTCGC-3'. This involved the following temperature cycle (Techne Genius, Chicago, IL, USA): 90°C, 3 minutes; 94°C for 1 minute, 52°C for 1 minute, 72°C for 30 seconds, repeated 30 times, and 72°C for 10 minutes. The MKα template was used as a positive control.

## Results

### Theoretical model

The model predicted an HGT frequency of less than 1.14 × 10^-16 ^per 100,000 generations at the 99% certainty level. The model was iterated twenty times, with the mean of the ten highest outputs evaluated at the 99% certainty level.

### Laboratory studies

#### Study 1

Results were uniform for plating of aliquots for all concentration ratios and all intervals. Growth of transformed *R. rhodnii *was visible on both BHI agar plates and BHI agar with kanamycin added at 50 μg/mL. Growth of *G. rubropertinctu*s was present on BHI agar but not on BHI agar with kanamycin added at 50 μg/mL.

#### Study 2

**Figure 2:**
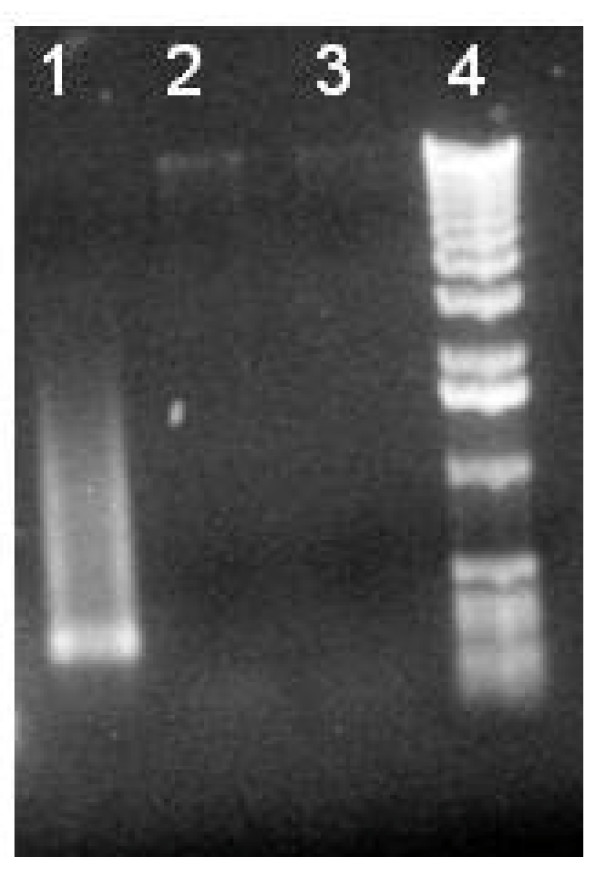
**PCR analysis of nymph gut contents for MKα region of pRrMDWK6: positive control (lane 1), gut contents of *R. prolixus *nymphs, (lanes 2 and 3), and DNA ladder, (lane 4)**. The MKα region was not present in gut contents of *R. prolixus *nymphs, indicating failure of gene transfer to wild-type *R. rhodnii*.

After 26 days of incubation, gut contents from all 71 nymphs showed growth on BHI agar at concentrations of 10^6 ^- 10^8 ^CFU/mL. No growth was detected on BHI with kanamycin from any of the 71 nymphs, indicating failure of transfer of the pRrMDWK6 plasmid. PCR analysis for the MKα region of pRrMDWK6 was negative for all 71 nymphs (Figure 2).

## Discussion

The transfer frequency predicted by our model is less than the average estimated mutation frequency in bacteria, 10^-1 ^per 1,000 generations. Lawrence and Roth hypothesize transfer frequency of a putatively fitness-neutral gene would have to be greater than mutation frequency of the recipient bacteria to be maintained in that organism [31]. This suggests that even if HGT were to occur between *R. rhodnii *and *G. rubropertinctus*, the transgene would likely not persist in the recipient organism.

The model presented in this study was developed to address risk assessment concerns associated with release of a genetically modified bacteria. The HGT frequency predicted would not vary significantly under conditions accompanying a large-scale release of the genetically modified bacteria. In this model ecological phenomena such as succession communities are subordinate to considerations attending gene integration such as fitness contribution and homology.

This taken into account, the model presented here has important limitations. The event of interest, horizontal transfer of a transgene not associated with a mobile element, has never been reported in any study. In adhering to the conventions of risk assessment, we have selected parameters for the model with the intention of predicting the upper limit of probability for a presumed adverse event. While crucial parameter values such as the rate of gene transfer and the percentage of donor bacterium genes conferring fitness advantage on the recipient bacterium are almost certainly elevated and the extent of homology necessary for integration is almost certainly lower than that required for integration in the environment, selecting what may be contrived parameters has the advantage of making possible the modeling of an exceedingly rare event.

In addition, while the model predicts a very low frequency of HGT over 100,000 generations, it is extremely unlikely that a fitness-neutral transgene would persist in a population for 100,000 generations. Previous studies indicate non-retention of the transgene in genetically modified bacteria is far more likely than HGT over this time period [12,32].

Our laboratory studies found no transfer of a foreign gene to wild-type *R. rhodnii *or *G. rubropertinctus*. The experiments exaggerated factors promoting HGT to a degree not attainable in the environment. This exaggeration extends to every parameter implicated in HGT, including the engineered bacterium. *R. rhodnii *used in these studies was transformed with an episomal plasmid, pRrMDWK6, whereas transformation of *R. rhodnii *for field release will involve use of an integrated element, a construct less amenable to HGT. In a 2003 study we demonstrated that a marker gene incorporated in *R. rhodnii *via an integrated element remained stable in culture for over 100 generations [23].

The marker gene used in this study coded for kanamycin resistance, a selective advantage under experimental conditions. Transfer of putatively fitness-neutral transgenes, such as those encoding cecropin A or transmission blocking single-chain antibodies, would be less favored. In these experiments, the marker gene was presented in a form (naked DNA) and in concentrations not attainable in the environment.

The bacteria were incubated in an enriched nutrient environment at very high rates of agitation (220 rpm). Bacterial mixing due to contractions of the arthropod gut occurs but not at such frequency. Further, bacterial populations in the gut lumen of *R. prolixus *reach a maxima of 10 ^8 ^CFU/mL following a blood meal, declining rapidly to an equilibrium concentration of approximately10 ^5 ^CFU/mL. The exaggerated bacterial concentrations featured in these studies would not be found in the environment.

The time period (168 hours) used in this study and intervals of collection were selected so as to evince transfer occurring through all three HGT modalities: transformation, transduction, and conjugation. The absence of tHGT under the grossly exaggerated conditions in these experiments argue strongly against tHGT occurring subsequent to environmental release of transformed *R. rhodnii*.

These thoroughly contrived experiments were designed to assess tHGT under extremely favorable conditions and yielded uniformly negative results. Nevertheless, some considerations for future studies remain. The putative recipient bacterium in this study, *G. rubropertinctus *was selected for its taxonomic relationship to *R. rhodnii *and environmental co-location. However, this study did not include *E. coli*, the bacterium whose genome contains the greatest percentage of horizontally acquired genes [8]. Since the generation time of *E. coli *is approximately 30 times faster than that of *R. rhodnii*, co-incubation studies of HGT are very difficult. In the future, use of other bacteria known for acquiring high percentages of genes horizontally may be considered.

It is also possible that other environmental bacteria, as independent populations or consortia, may act to promote gene transfer. Results from previous studies argue against this, showing highest rates of HGT under sterile conditions [18,28]. Limiting incubations to a potential donor and recipient bacterium pair prevents evaluation of the effect of such a microbial environment.

In addition, this experiment assessed tHGT over a 168-hour period. Absence of gene transfer over this time period under highly exaggerated conditions strongly suggests that likelihood is low. Further, the theoretical model cited above permits assessment over a time period - 100,000 generations - impractical for a laboratory experiment and obviates difficulties associated with detection of rare events. Because this model simulates HGT in the environment, it is therefore constrained in the extent to which its parameters can favor tHGT. In contrast, the laboratory experiments described above are not constrained and feature parameters whose exaggeration and coincidence would not occur in the environment. Results from this risk assessment may be extrapolated to the level of community ecology. Together, the uniformly negative results from these studies argue that the probability of tHGT occurring following environmental release of CRUZIGARD is vanishingly small.

## Conclusions

A mathematical model has been developed to predict the probability of horizontal gene transfer (HGT) between an engineered symbiotic bacterium used for paratransgenic control of vector-borne Chagas disease and a closely related environmental bacterium. The model treats HGT as a composite event, the probability of which is determined by the joint probability of three independent events: gene transfer through the modalities of transformation, transduction, and conjugation. Genes are represented in matrices and Monte Carlo method and Markov chain analysis are used to simulate and evaluate environmental conditions. The model is intended as a risk assessment instrument and predicts HGT frequency of less than 1.14 × 10^-16 ^per 100,000 bacterial generations. Initial laboratory studies designed to exaggerate the occurrence of HGT support the predictions of this model. The modeling exercise and laboratory-based simulation are necessary first steps in the development of a robust risk assessment framework for field implementation of paratransgenic control for Chagas disease. Additional studies, including assessment of HGT under field conditions and evaluation of HGT within microbial consortia that are found in nature, are required; results of this study suggest that HGT is a negligible consideration in risk assessment of genetically modified *R. rhodnii *released for control of Chagas disease.

## Abbreviations

HGT: Horizontal gene transfer; tHGT: transgene horizontal gene transfer; CFU: colony forming units; PBS: phonsphate buffered saline; PCR: polymerase chain reaction

## Competing interests

The authors declare that they have no competing interests.

## Authors' contributions

SM designed and performed the study. VSR and RVD managed, analyzed, and interpreted the data, and prepared the manuscript. All authors read and approved the final manuscript.
